# Nocardia Infection Complicated by Severe Hyponatremia in a Renal Transplant Patient

**DOI:** 10.7759/cureus.103038

**Published:** 2026-02-05

**Authors:** Saif Khan, Zishan Nasir, Syed Hidayat Ali Shah, Fahad Zamir, Nour Hani Jaouni

**Affiliations:** 1 Medicine, Hamad Medical Corporation, Doha, QAT; 2 Medicine, Weill Cornell Medicine-Qatar, Doha, QAT

**Keywords:** immunocompromised host, kidney transplantation, nocardia infection, opportunistic infections, pulmonary nocardiosis

## Abstract

Patients with solid organ transplants are at high risk for opportunistic infections due to the use of potent immunosuppressive drugs. Nocardiosis is a Gram-positive, aerobic actinomycete infection that is uncommon but can be serious in solid organ transplant recipients if not treated promptly. It often presents pulmonary or disseminated disease. Nocardia can infect immunocompromised patients mainly by airborne transmission. We recently encountered a challenging case of pulmonary nocardiosis with severe hyponatremia in a renal transplant recipient. We chose to write this case to highlight the association of pulmonary nocardiosis with severe hyponatremia in a renal transplant recipient, as nocardiosis rarely presents with profound electrolyte disturbances.

## Introduction

The widespread use of immunosuppressive therapy in kidney disease has been accompanied by an increase in infectious complications. Nocardia is an environmental organism commonly present in soil and stagnant water that causes opportunistic infections, predominantly affecting immunocompromised individuals [1]. Although nocardiosis remains a relatively rare opportunistic infection, it carries the potential for substantial morbidity and mortality in immunocompromised hosts. Among solid organ transplant recipients, the reported incidence of Nocardia infection ranges from 0.4% to 3.6% [2]. In renal transplant recipients, nocardiosis most often presents as pulmonary disease, with central nervous system and cutaneous being other common sites of involvement [3]. Nocardiosis carries a high fatality risk, estimated between 20% and 35%, with outcomes significantly worse when the infection disseminates or involves the central nervous system in renal transplant patients. Diagnosing nocardiosis in renal transplant patients is challenging due to its nonspecific clinical presentation and resemblance to other opportunistic infections. Symptoms such as fever, cough, and dyspnea often mimic bacterial pneumonia or tuberculosis [4]. Owing to the slow culture growth of Nocardia, early recognition depends largely on a high index of suspicion informed by clinical manifestations and radiological findings, which may be lifesaving [5]. We present this case to highlight the rarity of pulmonary Nocardia infection complicated by severe hyponatremia due to syndrome of inappropriate antidiuretic hormone secretion (SIADH) in a renal transplant recipient.

## Case presentation

**Figure 1:**
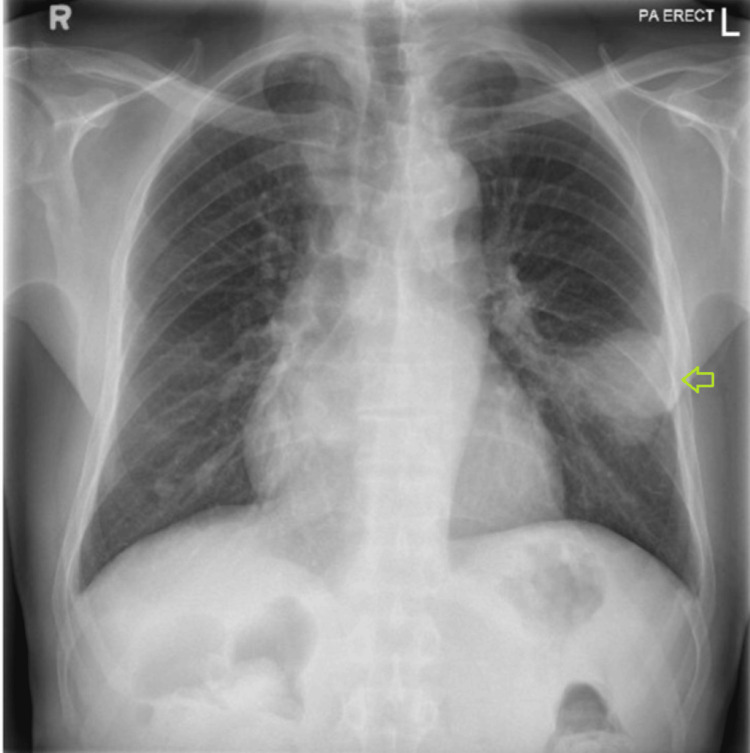
Posterior-anterior chest X-ray showed non-homogeneous opacity in the mid-lower zone of left lung (green arrow).

**Figure 2:**
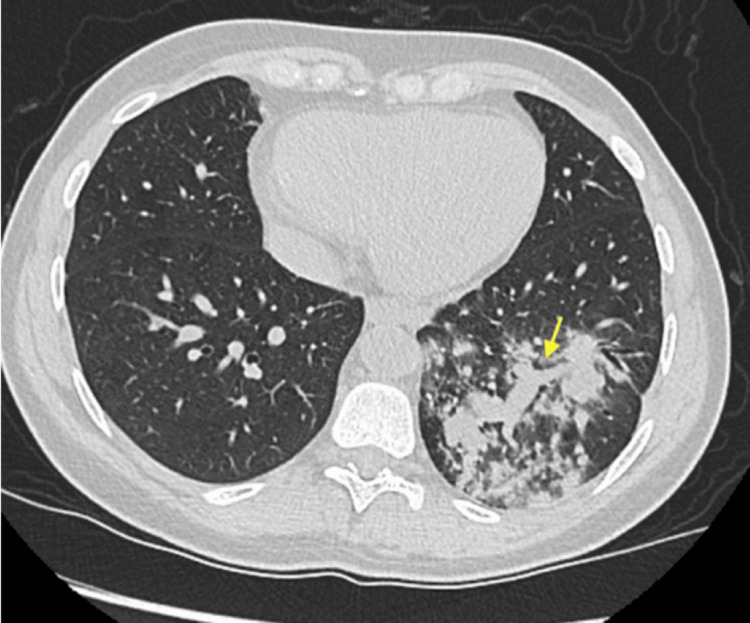
Spiral chest computed tomography (CT) demonstrated a cavitating lesion within an area of dense left lower lobe consolidation (yellow arrow).

**Figure 3:**
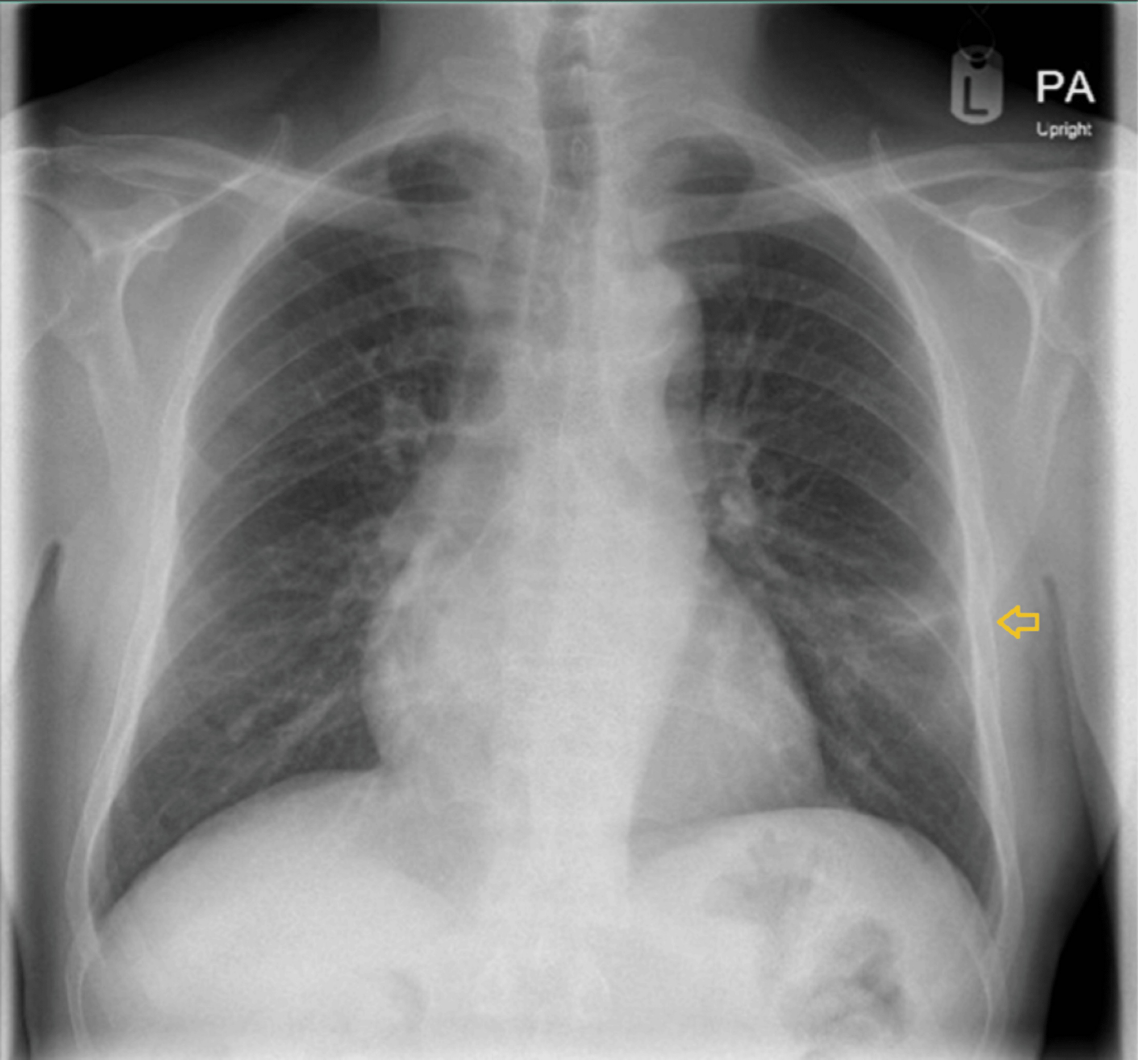
Follow-up X-ray after one month of treatment showed reduction in the density of left lower zone opacity (orange arrow).

Our patient is a 68-year-old man of Indian origin with end-stage renal disease secondary to long-standing diabetes mellitus who underwent a deceased-donor kidney transplant in December 2022. His post-transplant course was complicated by chronic allograft dysfunction, with a baseline serum creatinine of approximately 2.0 mg/dL. Induction immunosuppression consisted of antithymocyte globulin (ATG), and maintenance therapy included tacrolimus, mycophenolate mofetil, and prednisolone. He had completed one year of Pneumocystis jirovecii pneumonia prophylaxis with trimethoprim-sulfamethoxazole (TMP-SMX). The patient had been recently discharged following hospitalization for community-acquired pneumonia involving the left lower lobe, accompanied by hyponatremia. Physical examination at that time revealed crackles over the left lung base. Chest radiography demonstrated a left lower zone anterior-lateral segment consolidation measuring approximately 6.5 × 5 × 5 cm, abutting the oblique fissure and pleural surface, extending toward the hilar region, and containing internal air bronchograms with areas of hyperenhancement suggestive of exudative changes or necrosis (Figure 1). Computed tomography of the chest confirmed consolidation in the anterior-lateral segment of the left lower lobe, consistent with an infective etiology (Figure 2). Follow-up imaging was recommended to document resolution and exclude underlying pathology (Figure 3). Seven days after discharge, he was referred by his nephrologist from the outpatient clinic to the emergency department with recurrent fever, productive cough, and dyspnea of one week's duration. Three days prior to admission, he developed confusion and gait instability. On presentation, he was febrile (38.1°C) and hypertensive (150/90 mmHg). Neurological examination revealed disorientation, mild ataxia, and no focal deficits. His laboratory investigations were notable for leukocytosis (19,000 cells/µL). Blood chemistry revealed severe hyponatremia (serum sodium 114 mmol/L) associated with elevated serum creatinine (2.1 mg/dL). Further evaluation of the hyponatremia demonstrated low serum osmolality with inappropriately elevated urine osmolality (376 mOsm/kg) and euvolemia, fulfilling the diagnostic criteria for SIADH. The patient’s laboratory findings are summarized in Table 1.

**Table 1:** Laboratory results.

Test	Patient’s Values	Reference Range
Creatinine (Cr)	2.1 mg/dL	0.6-1.2 mg/dL
Blood urea nitrogen (BUN)	10 mg/dL	7-20.0 mg/dL
Sodium	114 mmol/L	135-146 mmol/L
WBC	19.5x3/microL	4-10x3/microL
Serum osmolality	271 mOsm/kg H2O	N = 275-295 mOsm/kg H2O
Urine osmolality	376 mOsm/kg H2O	N = 100-1200 mOsm/kg H2O

**Figure 4:**
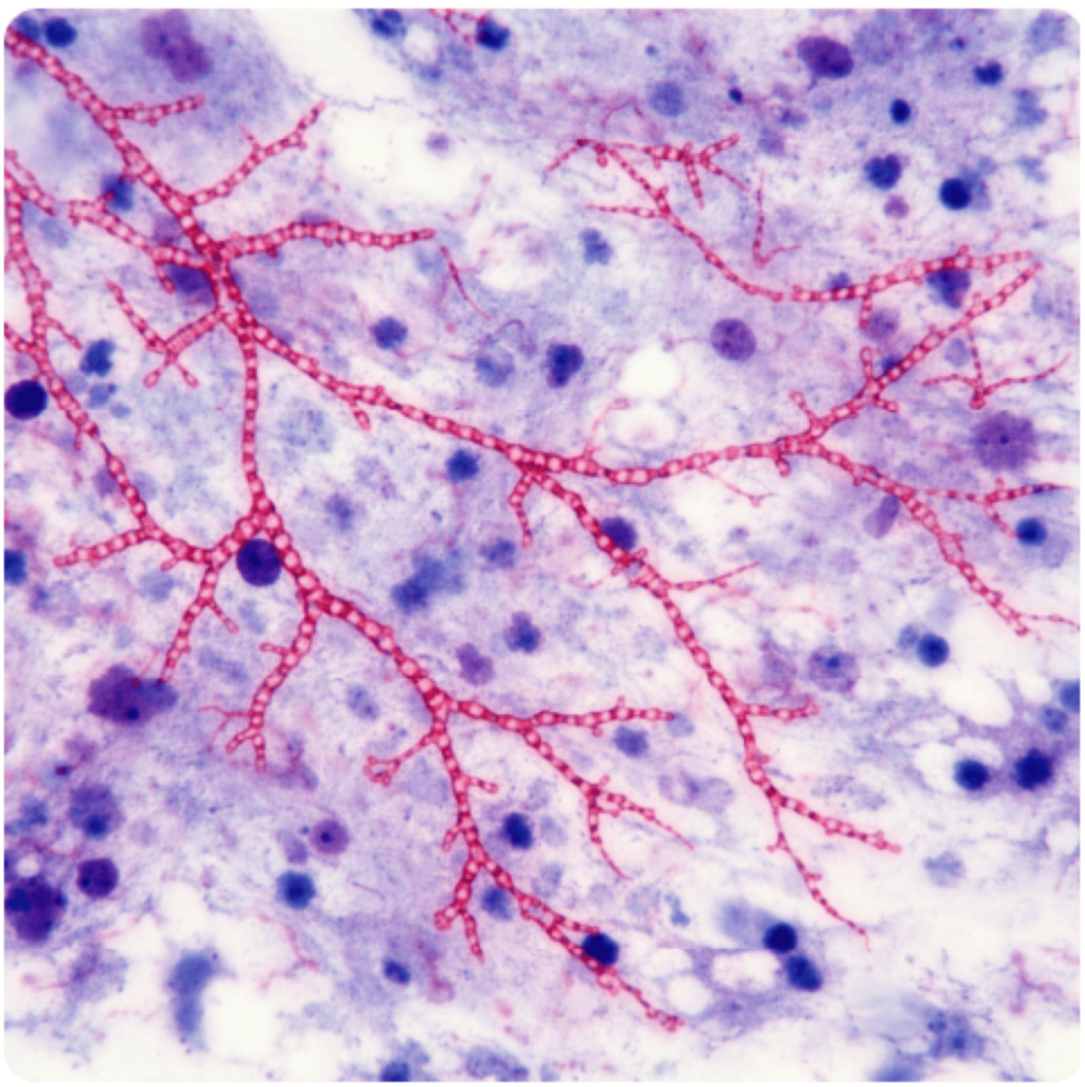
Beaded, branching filamentous organisms were identified in the bronchial lavage and were weakly acid-fast on Kinyoun staining, findings consistent with Nocardia species.

The patient was managed with close monitoring in a high-dependency setting. Given the severity of hyponatremia, treatment was initiated with careful correction of serum sodium using controlled intravenous hypertonic saline, alongside strict fluid balance monitoring. Serial serum sodium measurements were obtained to ensure a gradual rise in sodium levels and to avoid overly rapid correction. Given his immunocompromised status and persistent pulmonary consolidation, bronchoscopy was performed. Bronchoalveolar lavage revealed beaded, branching filamentous organisms that were weakly acid-fast on Kinyoun staining, consistent with Nocardia species (Figure 4). Empiric therapy with TMP-SMX was initiated, and immunosuppression was modified with dose reduction of tacrolimus and temporary reduction of mycophenolate. Subsequent sputum cultures confirmed Nocardia species resistant to TMP-SMX but susceptible to imipenem, amikacin, and linezolid. Antimicrobial therapy was adjusted accordingly, with induction therapy followed by step-down oral treatment using linezolid and minocycline, respectively. The patient demonstrated marked clinical improvement, with resolution of fever and neurological symptoms. Serum sodium normalized and SIADH resolved. He was discharged on hospital day 14 with a planned total antimicrobial treatment duration of 12 months. At one-month follow-up, he remained clinically well with stable graft function, normal serum sodium, and radiographic resolution with significant reduction in the density of the left lower zone opacity (Figure 3). Tacrolimus and mycophenolate doses were subsequently readjusted. 

## Discussion

**Figure 5:**
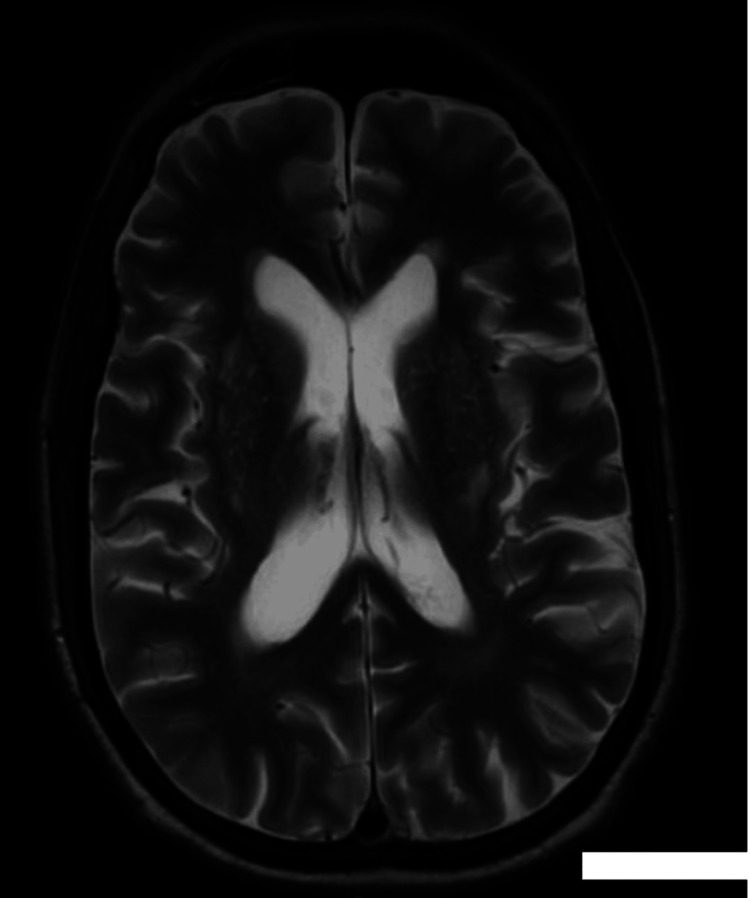
MRI brain - unremarkable.

Fungal infections are not uncommon in organ transplant recipients due to chronic immunosuppression [3]. SIADH is a very rare complication of nocardial infection, making this case especially unique. A review of the literature reveals only a handful of reported cases associating Nocardia infection with SIADH, particularly in renal transplant recipients. In the literature, only three cases of nocardiosis with severe hyponatremia have been documented. Crucially, the presence of SIADH in a patient with nocardiosis may serve as an important clue to central nervous system involvement, although pulmonary disease can similarly trigger SIADH. The mechanism involves increased antidiuretic hormone (ADH) release due to inflammatory cytokines or direct hypothalamic stimulation. Diagnosis of nocardiosis in renal transplant patients is often delayed due to its nonspecific and subtle clinical presentation, which in turn postpones timely initiation of therapy and frequently results in increased morbidity and mortality. Hyponatremia in renal transplant recipients is often attributed to medications or renal dysfunction. New-onset, unexplained hyponatremia in a patient with nocardiosis should prompt an active search for occult central nervous system involvement [6]. The profound hyponatremia in our patient raised suspicion for CNS involvement by Nocardia, as no other cause for SIADH was identified, prompting an early brain MRI (Figure 5). The initial differential diagnosis for his acute confusional status included metabolic encephalopathy versus a structural process. Posterior reversible encephalopathy syndrome (PRES) was briefly considered due to the MRI findings. Notably, after appropriate therapy was instituted, our patient’s serum sodium gradually normalized and his mental status improved in parallel, further supporting the linkage between treating the Nocardia infection and resolution of SIADH, as has been documented in other cases. Once we confirmed Nocardia infection in our patient, our attention turned to management. Therapy was initiated with intravenous TMP-SMX and continued with oral therapy using one double-strength tablet of the same medication twice a day.

## Conclusions

Nocardiosis is a rare but important opportunistic infection in renal transplant recipients that can be life-threatening. This case is novel in that Nocardia pneumonia manifested primarily as severe hyponatremia due to SIADH, without the classic expectation of disseminated disease. It underlines the need for vigilance for unusual presentations of infections in immunosuppressed patients. Early diagnosis (via invasive sampling and appropriate stains/cultures) and tailored therapy are crucial. Clinicians should remember that pneumonia-related SIADH is reversible with treatment of the underlying infection, and in cases of Nocardia, addressing the infection can lead to resolution of even severe hyponatremia. Furthermore, transplant physicians must balance infection management with modulation of immunosuppression and be alert to complications such as PRES. This case adds to the limited literature on nocardiosis-associated SIADH and demonstrates a successful outcome with multidisciplinary care. Awareness of such rare presentations can facilitate timely diagnosis and improve outcomes in similar scenarios in the future.
